# Possible association of pyogenic liver abscess with an inflamed sigmoid colonic lipoma: a case report and literature review

**DOI:** 10.3389/fmed.2026.1957072

**Published:** 2026-10-02

**Authors:** Khaireddine Dhouioui, Mohamed Abu Awad, Mohamed Hedi Mraidha, Soumaya Mrabet, Dorra Chiba, Raida Harbi, Elhem Ben Jazia, Moncef Mokni, Sabri Youssef

**Affiliations:** 1 Department of Gastrointestinal Surgery, Farhat Hached University Hospital, Faculty of Medicine, University of Sousse, Sousse, Tunisia; 2 Department of Gastroenterology, Farhat Hached University Hospital, Faculty of Medicine, University of Sousse, Sousse, Tunisia; 3 Department of Pathology, Farhat Hached University Hospital, Faculty of Medicine, University of Sousse, Sousse, Tunisia

**Keywords:** colonic lipoma, *Klebsiella pneumoniae*, portal bacteremia, pyogenic liver abscess, sigmoid colon

## Abstract

**Background:**

Pyogenic liver abscess (PLA) is a severe infectious condition that is often associated with an identifiable biliary or gastrointestinal source. Benign colonic lesions are rarely considered during the etiological evaluation of PLA.

**Case presentation:**

We report the case of a 47-year-old man admitted with febrile diarrhea. Contrast-enhanced computed tomography (CT) demonstrated confluent hepatic abscesses involving segments II, III, and IV, measuring 10 cm × 9 cm × 7 cm, without a CT-detectable biliary source. The etiological investigation identified an approximately 2 cm inflammatory sigmoid lesion that was initially considered suspicious for malignancy. Laparoscopic oncologic segmental low sigmoid colectomy was performed because malignancy could not be confidently excluded based on superficial endoscopic biopsies. Histopathological examination confirmed a 1.2 cm submucosal lipoma composed of mature univacuolar adipocytes without cytologic atypia, necrosis, or abscess formation. The colonic wall directly opposite the lipoma showed acute neutrophil-rich inflammation extending from the mesocolic/subserosal tissues through the muscularis propria, together with focal superficial epithelial abrasion/erosion of the overlying mucosa and exposure of the lamina propria. Deep or transmural mucosal ulceration, ischemic necrosis, intramural abscess, microorganisms, perforation, and diverticular disease were not demonstrated in the available histopathological sections. Blood cultures were negative. Culture of the hepatic abscess aspirate isolated *Klebsiella pneumoniae.* The colonic lesion itself was not cultured, and no microbiological or molecular comparison with the hepatic isolate was performed. No phenotypic or molecular testing for a hypervirulent *K. pneumoniae* phenotype was conducted.

**Conclusion:**

This case describes a possible association between PLA and an inflamed sigmoid submucosal lipoma with focal superficial epithelial abrasion/erosion. A causal relationship cannot be established from the available evidence. The clinical implication is that colonic evaluation, including colonoscopy when clinically appropriate, may be considered in otherwise unexplained PLA, particularly when no biliary or other common source is identified. This single case does not establish small colonic lipomas as infectious sources of PLA.

## Introduction

Pyogenic liver abscess (PLA) is a serious infectious condition that may be life-threatening. Its reported incidence varies considerably by geographic region, ranging from approximately 1–3 cases per 100,000 population per year in several Western cohorts to more than 17 per 100,000 in some Asian series. An increase in reported incidence has been observed over recent decades (1–3).

Common causes include biliary tract disease, intra-abdominal infectious foci, and hematogenous dissemination via the portal venous system (1–3). Biliary tract disease is now regarded as the most common identifiable source of PLA, having surpassed appendicitis and other intra-abdominal infections in contemporary series (4). Colonic lesions—whether benign or malignant—that disrupt the mucosal barrier may provide a potential route for portal bacterial dissemination and subsequent hepatic abscess formation (5, 6).

Diverticulitis, colorectal carcinoma, and inflammatory bowel disease are among the typical colon-related causes of PLA (1, 3). Colonic lipomas are uncommon benign mesenchymal tumors, most often located in the submucosa. They are among the more frequent non-adenomatous colonic tumors. A subset of lesions larger than 2 cm can become symptomatic (7–9).

A structured literature search of PubMed/MEDLINE, Embase, and Google Scholar was performed on 1 September 2026 using combinations of the terms “Pyogenic liver abscess”, “liver abscess”, “colonic lipoma”, “colon lipoma”, “benign colonic lesion”, “colonic adenoma”, “tubulovillous adenoma”, “villous adenoma”, “colorectal neoplasia”, and “Klebsiella pneumoniae”. Titles and abstracts were screened for relevance, and potentially relevant articles were assessed by full-text review. Reports were considered eligible for inclusion in the literature summary if they described a pyogenic liver abscess occurring in association with a benign or premalignant colonic or closely related gastrointestinal lesion. Reference lists of relevant articles were also manually screened to identify additional reports not retrieved by the electronic searches. Based on this search and screening process, no previous report specifically describing a pyogenic liver abscess associated with a colonic lipoma was identified. Selected relevant reports are summarized in Table 1. Several reports have described PLA in association with other benign colonic lesions, particularly adenomas (6, 10–12). A duodenal ulcerated lipoma has also been reported in association with a liver abscess (13). These observations support a plausible, but unproven, pathophysiological link between selected benign gastrointestinal lesions and PLA.

**Table 1:** Selected published reports of pyogenic liver abscess associated with benign or premalignant gastrointestinal lesions.

Report	Lesion	Site	Organism	Comment
Lai et al. 2006 (6)	Tubulovillous adenoma (2 cases)	Colon (one sigmoid)	*K. pneumoniae* in 1	No biliary source; association proposed
Leiba et al. 2003 (10)	Villous adenoma	Colon	Not specified	PLA as presenting clue
Tan et al. 2012 (12)	Tubulovillous adenoma	Colon	No organism isolated	Organizing cryptogenic abscess
Chen et al. 2012 (11)	Adenomatous polyps/focal adenocarcinoma in adenoma (*n* = 12)	Colon	Mixed	Series of PLA with colorectal neoplasia
Mifuji-Moroka et al. 2012 (13)	Ulcerated lipoma	Duodenum	Not specified	Closest published report involving a gastrointestinal lipoma and liver abscess
Present case	Inflamed submucosal lipoma (1.2 cm) with focal superficial epithelial abrasion/erosion	Sigmoid colon	*K. pneumoniae* (hepatic abscess only)	Colonic lesion not cultured; no microbiological or molecular comparison performed. Acute neutrophil-rich inflammation involving the mesocolic/subserosal tissues and extending into the muscularis propria, with focal superficial epithelial abrasion opposite the lipoma. Deep mucosal ulceration, ischemic necrosis, intramural abscess formation, microorganisms, and perforation or microperforation were not demonstrated

We report a case of PLA temporally associated with an inflamed submucosal sigmoid lipoma. Histology demonstrated acute neutrophil-rich inflammation extending from the mesocolic/subserosal tissues through the muscularis propria, together with focal superficial epithelial abrasion/erosion of the underlying lamina propria. Deep mucosal ulceration was not demonstrated on the available histopathological sections. The relationship is presented as a possible association rather than as a confirmed source of infection.

## Case presentation

A 47-year-old man with no notable medical history, no known diabetes mellitus, and no history of immunosuppressive therapy or chronic illness presented with a 4-day history of febrile diarrhea. On physical examination, the patient was febrile (38.3 °C) but hemodynamically stable, with a blood pressure of 120/80 mmHg and a respiratory rate of 17 breaths/min. He was fully oriented, with no altered mental status, and exhibited right upper quadrant tenderness without guarding or rebound tenderness. The remainder of the clinical examination was unremarkable.

Initial laboratory investigations revealed a marked inflammatory response, with leukocytosis (16,530/mm^3^), neutrophilia (13,200/mm^3^), elevated C-reactive protein (269 mg/L), and procalcitonin (2.4 ng/mL). Hemoglobin was 12.5 g/dL, platelet count was 184,200/mm^3^, serum creatinine was 74 µmol/L, total bilirubin was 15 µmol/L, direct bilirubin was 4 µmol/L, albumin was 35 g/L, blood glucose was 5 mmol/L, and international normalized ratio (INR) was 1. Hypokalemia was also noted (2.8 mmol/L). Liver and pancreatic enzyme testing showed aspartate aminotransferase (AST) 66 U/L (1.6 × N), alanine aminotransferase (ALT) 85 U/L (2.1 × N), gamma-glutamyl transferase (GGT) 77 U/L, alkaline phosphatase (ALP) 85 U/L (within the normal range), and lipase 20 U/L (within the normal range). HbA1c was not performed. Contrast-enhanced abdominal computed tomography (CT) demonstrated confluent hepatic abscesses involving segments II, III, and IV, with an overall size of 10 cm × 9 cm × 7 cm, without associated biliary abnormality (Figure 1).

**Figure 1:**
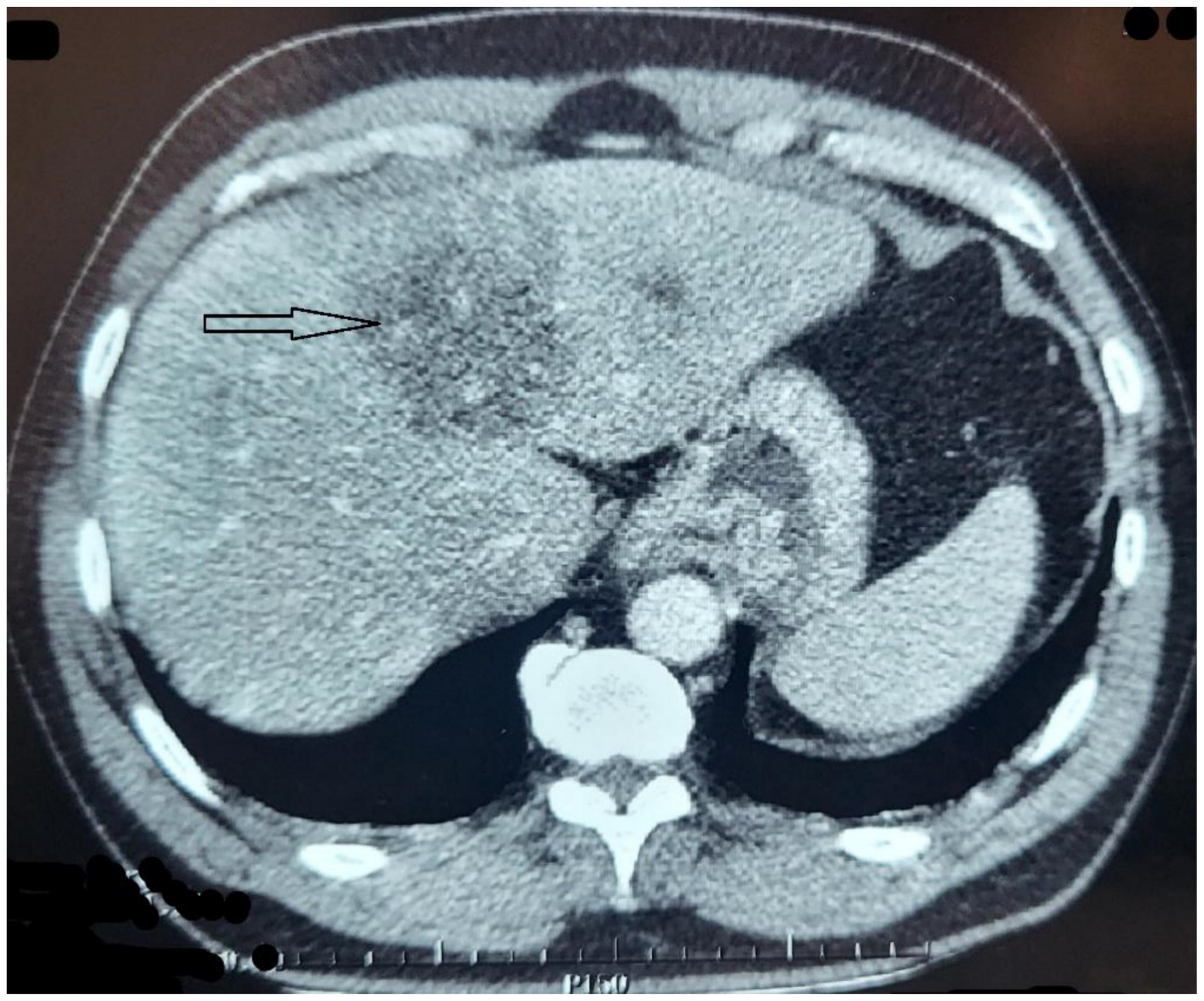
Contrast-enhanced CT showing confluent hepatic abscesses involving segments II, III, and IV (overall size 10 cm × 9 cm × 7 cm) (arrow), without biliary dilatation or an identifiable biliary source.

No intrahepatic or extrahepatic biliary dilatation, gallstones, or other CT-detectable biliary sources were identified. Abdominal ultrasonography performed to guide drainage confirmed the hepatic collections. Magnetic resonance cholangiopancreatography showed no biliary abnormality. Echocardiography revealed no evidence of an intracardiac or endovascular source of infection. Chest radiography showed no respiratory source of infection. Stool microbiological testing was negative, and serological testing for amoebiasis and hydatidosis was negative. HIV testing was also negative. Dental evaluation did not identify an infectious focus, and urinalysis was negative, with no evidence of a urinary source of infection.

The patient was admitted to a high-dependency unit and received intravenous fluid resuscitation, correction of hypokalemia, and empirical antibiotic therapy combining cefotaxime (1 g every 8 h) and metronidazole (500 mg every 8 h). Blood cultures yielded no growth. Antibiotic therapy was initiated before percutaneous drainage. Ultrasound-guided percutaneous drainage was performed 48 h after admission using a 14-Fr catheter, and the hepatic abscess aspirate was obtained at the time of drainage, after the initiation of antibiotic therapy. Approximately 100 mL of purulent material was initially drained. Daily drain output subsequently decreased to approximately 50 mL per day over the following 7 days. After 10 days of drainage, the patient showed clinical improvement with resolution of the systemic inflammatory response, and no further material was obtained through the catheter. The catheter was therefore removed.

Direct microscopic examination of the hepatic abscess aspirate did not reveal microorganisms, whereas subsequent culture isolated *Klebsiella pneumoniae*. The specimen was cultured on blood agar under aerobic and anaerobic conditions at 37 °C, with microbiological monitoring for up to 7 days. The isolate was susceptible to amoxicillin–clavulanic acid, amikacin, ceftazidime, ciprofloxacin, colistin, ertapenem, fosfomycin, cefoxitin, cefotaxime, gentamicin, imipenem, cephalexin, piperacillin–tazobactam, trimethoprim–sulfamethoxazole, and tigecycline, and resistant to ampicillin and ticarcillin. Blood cultures remained negative. No phenotypic or molecular testing for a hypervirulent *K. pneumoniae* phenotype was performed; therefore, the isolate cannot be classified as hypervirulent based on the available data.

Intravenous antibiotic therapy with cefotaxime and metronidazole was administered for 14 days, followed by a 7-day oral step-down regimen with amoxicillin–clavulanic acid. A follow-up abdominal CT scan performed 1 month later showed a favorable radiological response (Figure 2).

**Figure 2:**
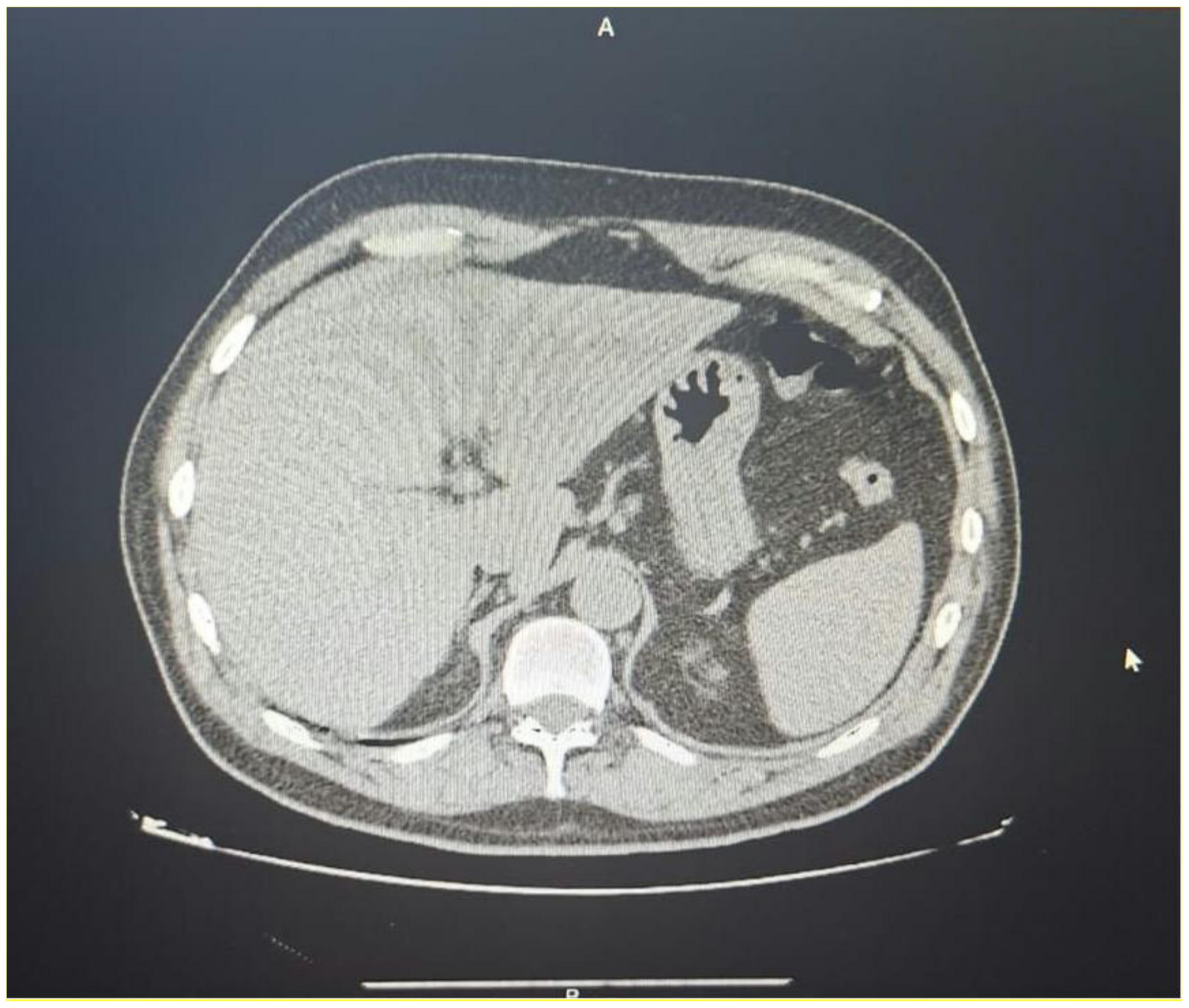
Follow-up axial contrast-enhanced abdominal CT obtained one month after initial presentation, following percutaneous drainage and antimicrobial therapy. The previously confluent abscesses in segments II-IV show marked radiological response.

As part of the etiological work-up, colonoscopy performed 3 weeks after symptom onset identified a broad-based, sessile, inflammatory polypoid lesion of the sigmoid colon measuring approximately 2 cm, which was considered suspicious for malignancy (Figure 3). No diverticula were noted in the sigmoid colon. Histopathological examination of endoscopic biopsies showed no evidence of tumor cells. A separate 5 mm sigmoid polyp was resected by snare polypectomy, and histological examination revealed low-grade dysplasia (Figure 4). The 5 mm polyp was distinct from the 2 cm inflammatory lesion and is not considered the source of the abscess.

**Figure 3:**
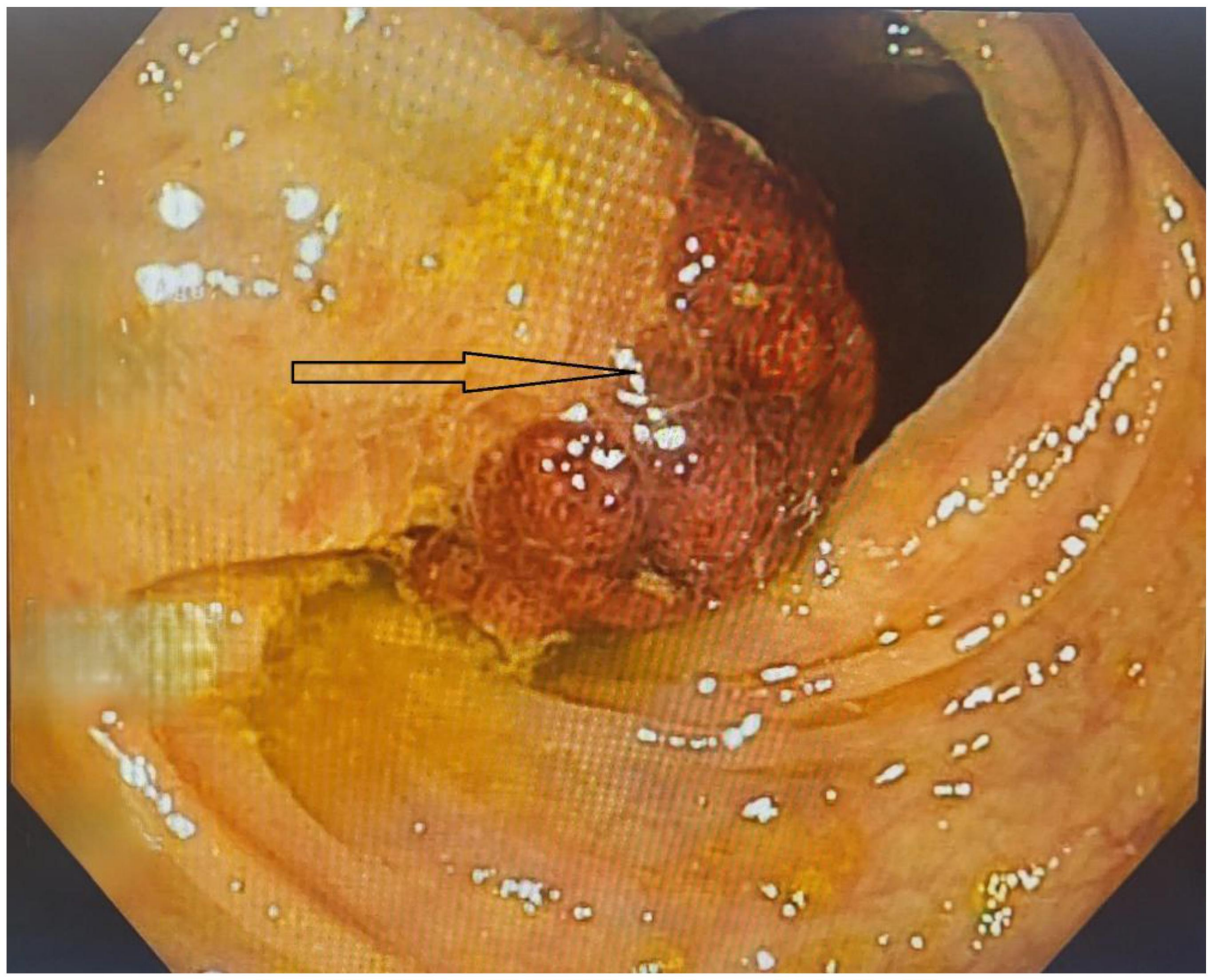
Colonoscopic view of a broad-based, sessile, inflammatory polypoid lesion of the sigmoid colon, measuring approximately 2 cm and showing features suspicious for malignancy (arrow). This endoscopic appearance prompted surgical resection after non-diagnostic superficial biopsies. The endoscopic appearance alone does not establish mucosal ulceration or infection of the underlying lipoma.

**Figure 4:**
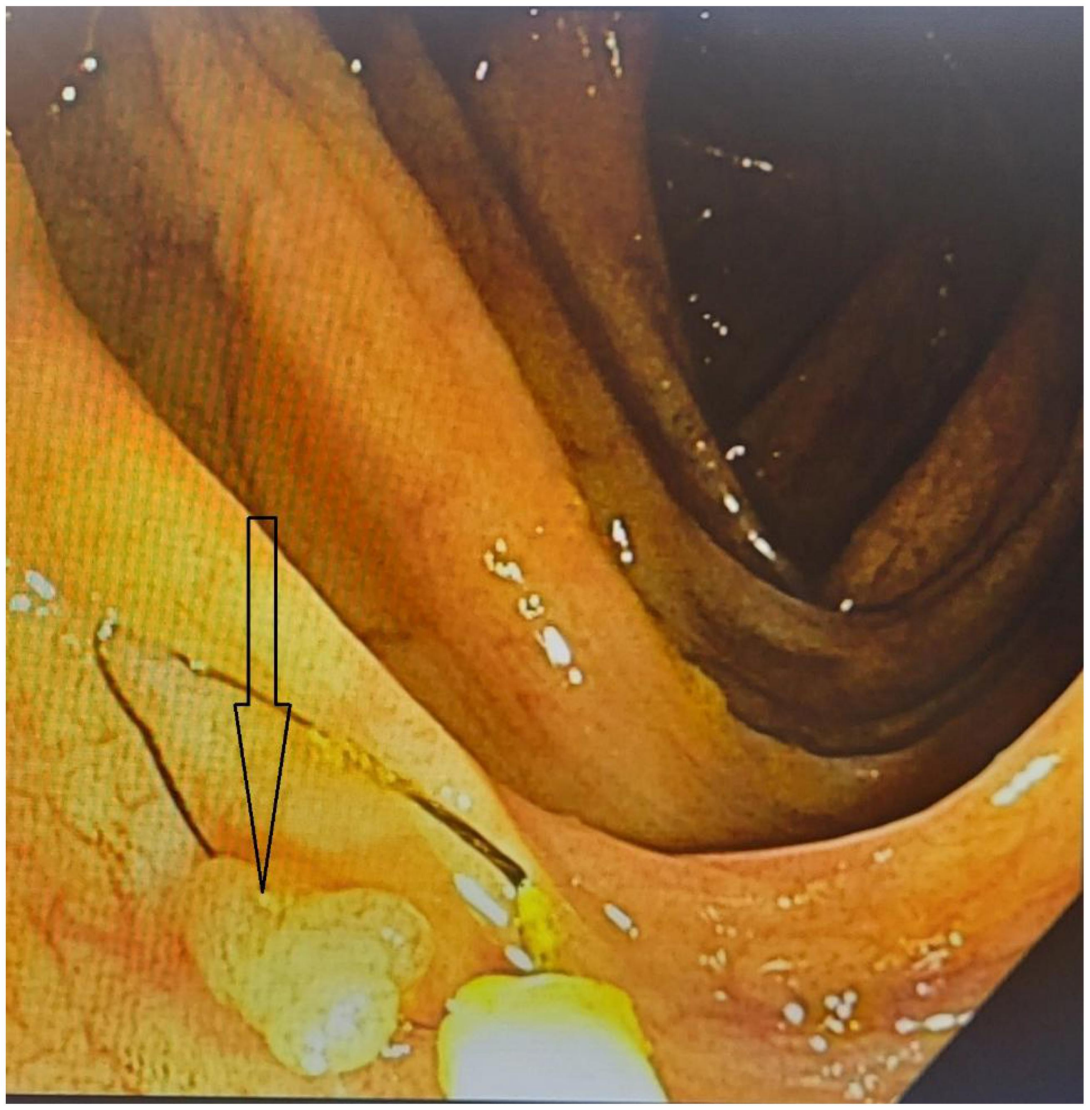
Separate 5 mm pedunculated sigmoid polyp resected by snare polypectomy, showing low-grade dysplasia. This polyp was distinct from the 2 cm inflammatory lesion and was not considered a likely source of the pyogenic liver abscess.

Endoscopic resection of the 2 cm lesion, endoscopic ultrasound, tattooing, and repeat deeper biopsy were not performed. Although the initial biopsies showed no malignant cells, they were superficial and considered insufficient to reliably exclude an underlying neoplasm due to the persistent suspicious endoscopic appearance. After a multidisciplinary discussion, a laparoscopic oncologic segmental low sigmoid colectomy was chosen as the definitive diagnostic and therapeutic approach rather than a limited local resection, because malignancy could not be confidently excluded preoperatively. The intraoperative appearance suggested a lipoma (Figure 5), but this finding was observed only after the surgical decision had been made and therefore did not influence the indication for surgery. This decision reflected diagnostic uncertainty regarding possible malignancy rather than evidence that the lesion represented an infectious focus. The postoperative course was uneventful, and the patient was discharged on postoperative day 6.

**Figure 5:**
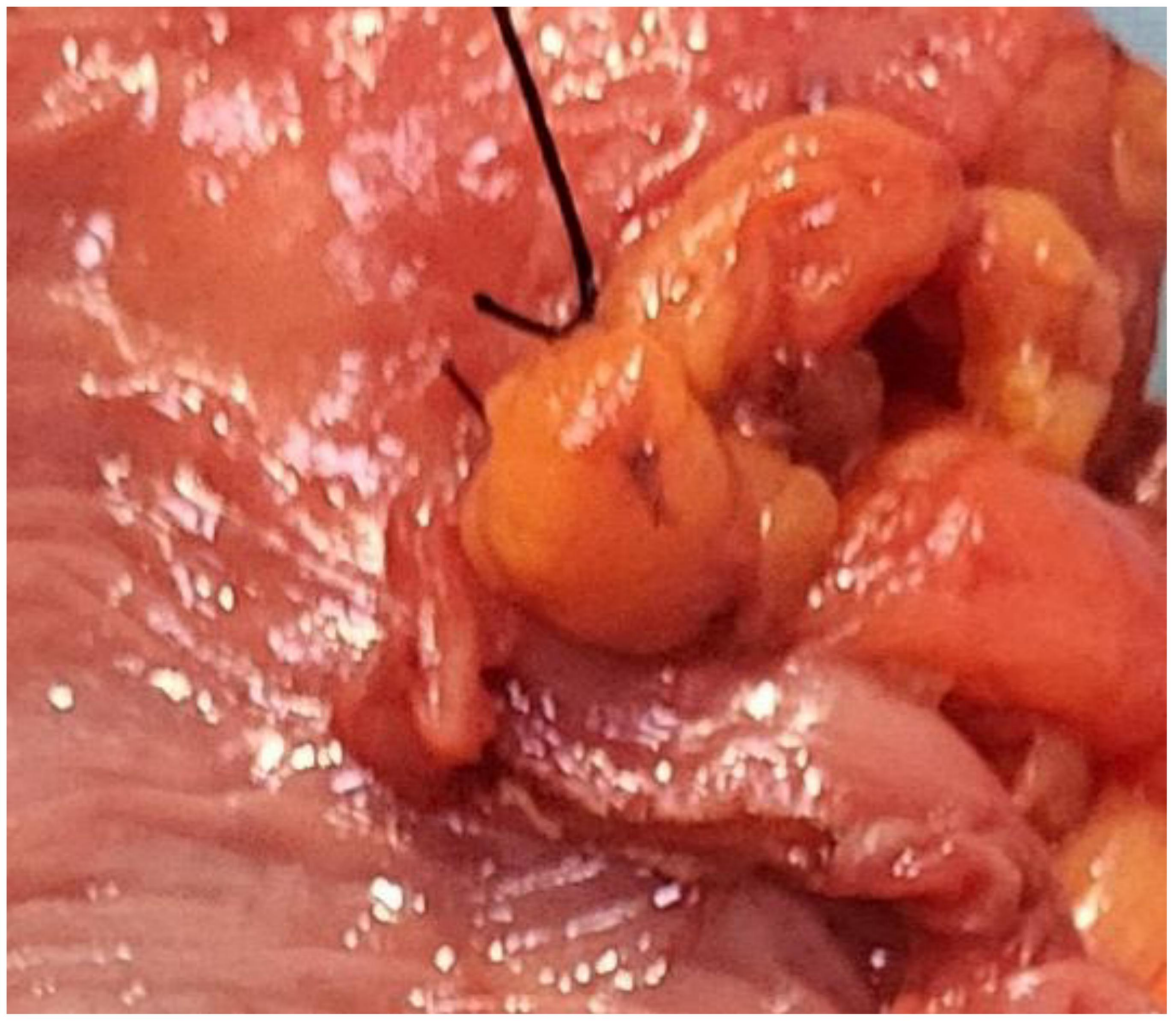
Gross examination of the opened resected sigmoid colon specimen showing a yellowish submucosal lipoma identified by a surgical stitch. The yellow cut surface is consistent with adipose tissue and corresponds to the mature lipoma confirmed histologically (Figure 6A).

Histopathological examination of the resected specimen confirmed a submucosal lipoma measuring 1.2 cm in greatest diameter, composed of mature univacuolar adipocytes separated by thin fibrous septa, without cytologic atypia, necrosis, or abscess formation (Figure 6A). Surgical margins and colorectal donuts were free of disease. Sections of the colonic wall taken directly opposite the lipoma showed transmural inflammation, with vascular congestion and a dense neutrophil-rich inflammatory infiltrate extending from the mesocolic/subserosal adipose tissue through the full thickness of the muscularis propria (Figure 6B). These findings support acute neutrophilic inflammation involving the full thickness of the bowel wall opposite the lesion and are compatible with the acute peritoneal reaction described in the original pathology assessment. Because a dedicated mesothelial-surface field is not available, acute serositis is regarded as consistent with that narrative rather than independently proven in the photomicrographs. The inflammatory changes indicate an active transmural process in the resected segment. They do not, by themselves, identify the lipoma as an infected mass or as the source of the hepatic abscess. The overlying colonic mucosa directly opposite the lipoma showed preserved crypt architecture and goblet-cell differentiation laterally, with focal abrasion of the surface epithelium and exposure of the underlying lamina propria centrally, together with a detached fragment of mature adipose tissue corresponding to the adjacent lipoma (Figure 6C). This appearance corresponds to a superficial epithelial abrasion/erosion rather than a deep or transmural ulcer, as the defect did not extend through the muscularis mucosae. No ischemic or coagulative necrosis, organized intramural or lipomatous abscess cavity, bacterial colonies or other microorganisms, perforation or microperforation tract, or diverticula/diverticulitis were identified in the available hematoxylin–eosin fields. No Gram, Giemsa, Periodic Acid-Schiff (PAS), or other special stains were performed, and the colonic lesion was not submitted for culture.

**Figure 6:**
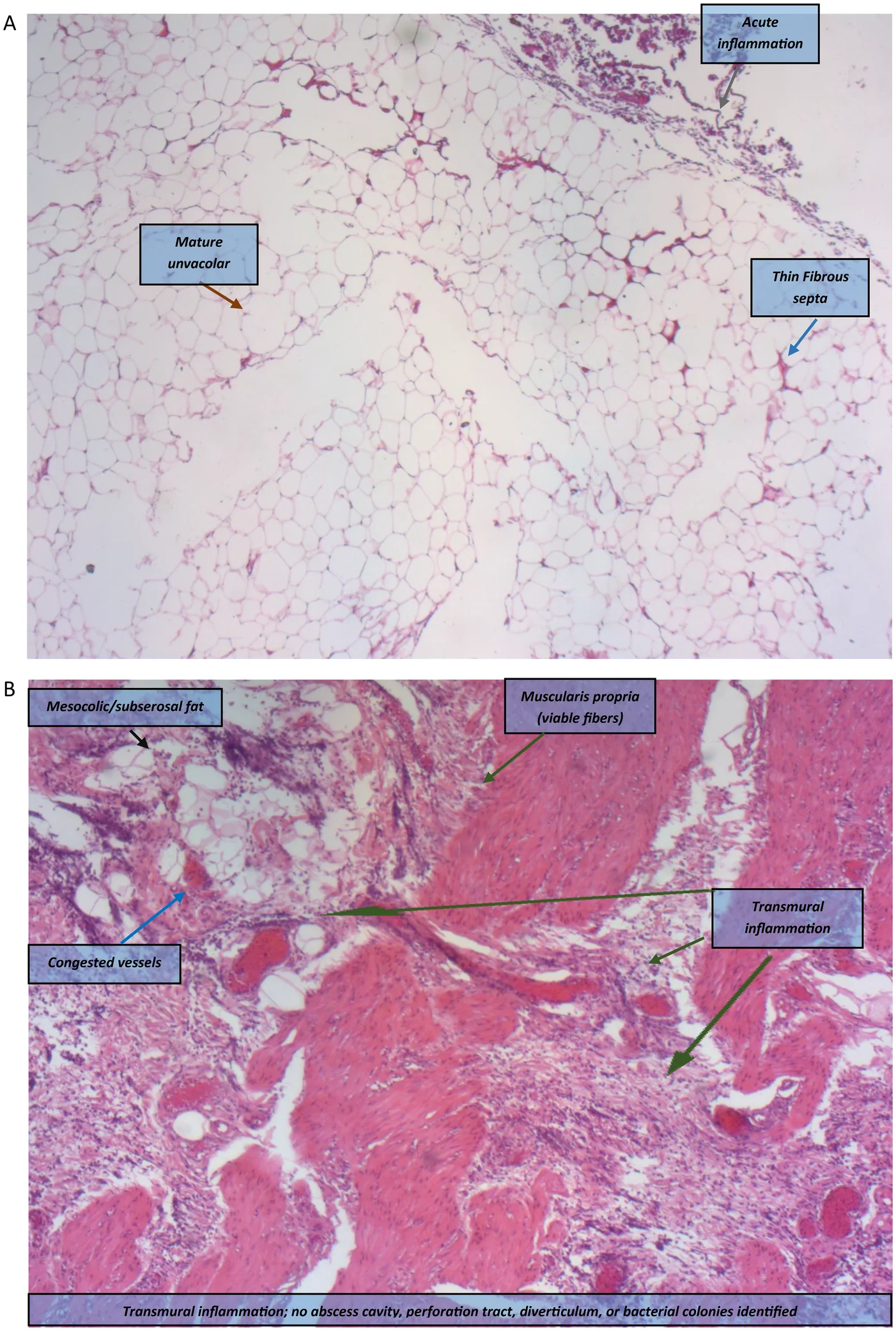

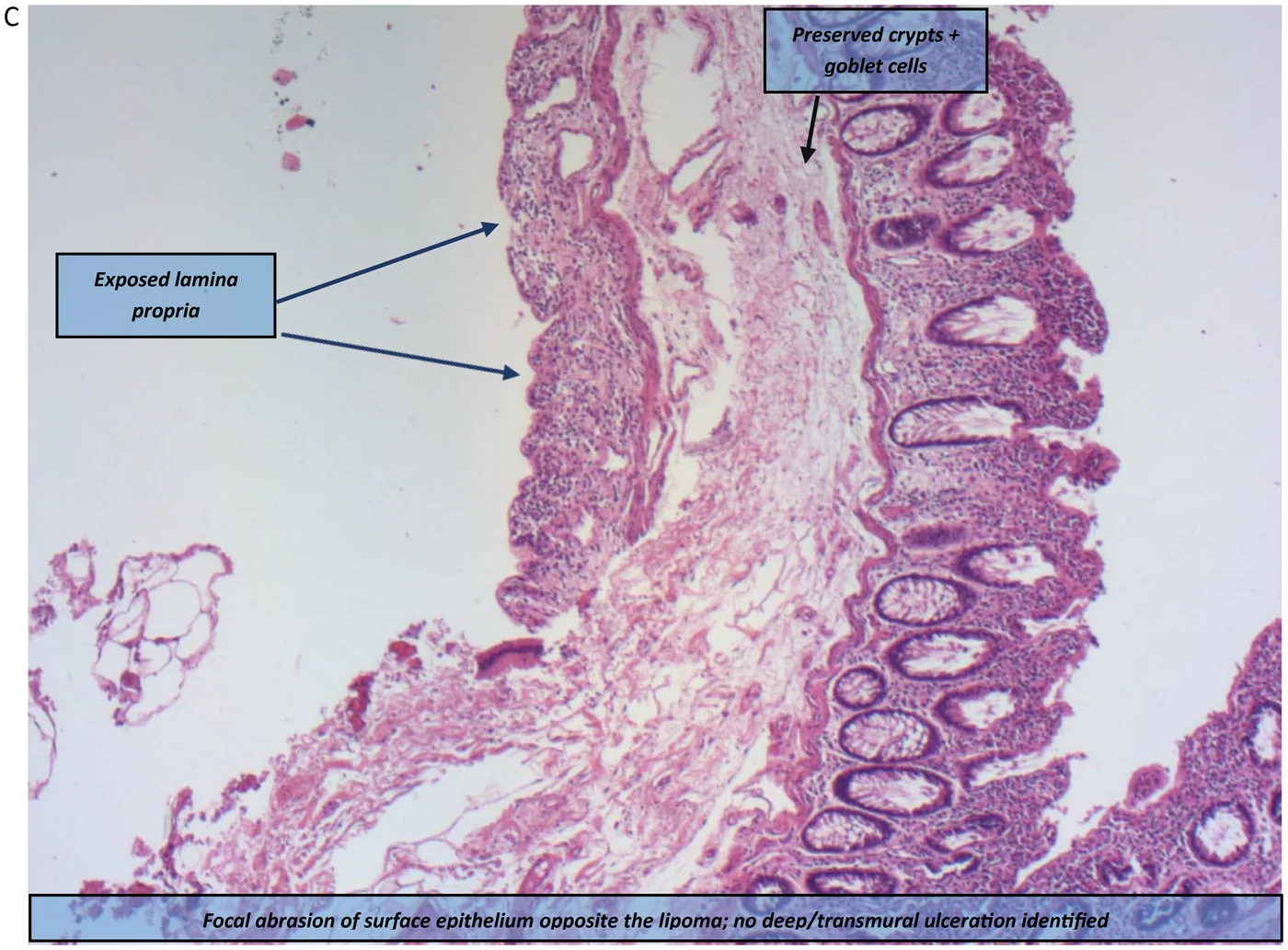
Histopathology of the resected sigmoid lesion (hematoxylin–eosin). **(A)** Submucosal lipoma composed of mature univacuolar adipocytes with small peripheral nuclei separated by thin fibrous septa, without cytologic atypia. No necrosis or abscess formation is identified in this field; a fragment of adjacent inflamed tissue is seen at the periphery (upper right). These histological features confirm the diagnosis of a mature submucosal lipoma. High-power magnification (×200); Scale bar, 50 µm. **(B)** Colonic wall directly opposite the lipoma showing transmural inflammation. Mesocolic/subserosal adipose tissue (upper left) is contiguous with congested vessels and a dense neutrophil-rich inflammatory infiltrate that extends into the muscularis propria, with inflammatory cells dissecting between individual muscle fibers (intramuscular inflammatory infiltrate). These findings support acute neutrophilic inflammation extending through the full thickness of the wall opposite the lesion and are compatible with the acute peritoneal inflammatory reaction described in the original pathology assessment. No organized abscess cavity, bacterial colonies, diverticulum, or perforation tract is identified in the field shown. High-power magnification (×400); Scale bar, 50 µm. **(C)** Colonic mucosa directly opposite the lipoma. On the right, crypt architecture is preserved with abundant goblet cells and an intact surface epithelium. Centrally, the mucosal fold shows focal abrasion of the surface epithelium with exposure of the underlying lamina propria, without evidence of deep or transmural ulceration, ischemic necrosis, or microorganisms. A detached fragment of mature adipose tissue, corresponding to the adjacent lipoma, is visible at the lower left margin of the field. High-power magnification (×200); Scale bar, 50 µm.

The lesion had been estimated endoscopically at approximately 2 cm. The difference between the endoscopic and pathological measurements may be explained by the broad-based inflammatory appearance of the lesion on endoscopy and tissue shrinkage following formalin fixation. The pathological measurement refers to the lipoma itself rather than the surrounding inflammatory or edematous tissue.

A follow-up colonoscopy performed one year later showed no abnormalities. The patient reported satisfaction with the treatment outcome and relief following recovery from the acute episode.

Table 2 presents the timeline of clinical events.

**Table 2:** Timeline of the clinical course.

Time point	Event
Days—4 to 0	Onset of febrile diarrhea, followed by hospital presentation 4 days after symptom onset.
Day 0—Admission	Fever 38.3 °C, blood pressure 120/80 mmHg, respiratory rate 17/min, fully oriented with no altered mental status, and right upper quadrant tenderness without guarding or rebound tenderness. Laboratory investigations showed leukocytosis (16,530/mm^3^) with neutrophilia (13,200/mm^3^), platelets 184,200/mm^3^, C-Reactive Protein (CRP) 269 mg/L, procalcitonin 2.4 ng/mL, hypokalemia (2.8 mmol/L), hemoglobin 12.5 g/dL, creatinine 74 µmol/L, albumin 35 g/L, glucose 5 mmol/L, and INR 1. AST was 66 U/L (1.6 × N), ALT 85 U/L (2.1 × N), GGT 77 U/L, ALP 85 U/L (normal), Total bilirubin 15 µmol/L, and lipase 20 U/L. Contrast-enhanced CT showed confluent hepatic abscesses involving segments II-IV, measuring 10 cm × 9 cm × 7 cm, without a CT-detectable biliary abnormality. Blood cultures were obtained and subsequently remained negative. Intravenous cefotaxime plus metronidazole was initiated.
48 h after admission	Ultrasound-guided percutaneous drainage was performed using a 14-Fr catheter. Approximately 100 mL of purulent material was aspirated initially. Direct bacteriological examination was negative, whereas subsequent culture grew *K. pneumoniae*, susceptible to cefotaxime and amoxicillin–clavulanic acid.
Days 1‒7 after drainage	Drain output progressively decreased to approximately 50 mL/day over the following 7 days. Clinical and biological improvement was observed.
Day 10 after drainage	No further material was obtained through the drain, with continued clinical improvement and resolution of the systemic inflammatory response. The drain was removed.
Days 1‒14 of treatment	Intravenous cefotaxime and metronidazole were continued for a total of 14 days, followed by a 7-day oral step-down regimen with amoxicillin–clavulanic acid.
Approximately 3 weeks after symptom onset	Colonoscopy revealed a broad-based, sessile, inflammatory sigmoid lesion measuring approximately 2 cm and suspicious for malignancy. No sigmoid diverticula were identified. Endoscopic biopsies showed no tumor cells. A separate 5 mm sigmoid polyp with low-grade dysplasia was removed by snare polypectomy.
After multidisciplinary review	Because the endoscopic appearance remained suspicious for malignancy and the biopsies were considered insufficient to exclude an underlying neoplasm, laparoscopic oncologic segmental low sigmoid colectomy was performed. Gross examination revealed a submucosal lipoma. The patient was discharged on postoperative day 6.
Final pathology	The resected lesion was a 1.2 cm submucosal lipoma associated with an acute peritoneal reaction and acute neutrophil-rich inflammation involving the mesocolic/subserosal tissues and extending into the muscularis propria, with focal superficial abrasion of the overlying surface epithelium. Deep mucosal ulceration, ischemic necrosis, intramural abscess formation, and microorganisms were not demonstrated in the available pathology material. Surgical margins were free.
1 month after initial presentation	Follow-up abdominal CT demonstrated favorable radiological response of the hepatic abscesses.
1 year	Surveillance colonoscopy showed no abnormalities.

## Discussion

Despite advances in imaging, image-guided drainage, and antimicrobial therapy, PLA remains a potentially life-threatening disease. Its epidemiological pattern has shifted over time, with biliary tract disease replacing appendicitis and other intra-abdominal infections as the leading identifiable cause. Even so, many cases remain cryptogenic despite extensive diagnostic evaluation (14). Bacterial translocation from the colon into the portal circulation is widely recognized as an important mechanism in the pathophysiology of PLA. Colonic lesions that compromise mucosal integrity—including colorectal cancer, large polyps, ulcerations, and diverticulitis—have been identified as potential infectious foci capable of causing PLA (5, 6).

In a retrospective study of cryptogenic pyogenic liver abscesses, Jeong et al. identified underlying colonic pathology, including polyps and ulcerated lesions, in nearly 25% of patients (5). More recent evidence has further supported this observation: *K. pneumoniae*, including hypervirulent strains in published series, has been associated with underlying colorectal neoplasia, and several studies and case reports have recommended colonoscopic evaluation in patients with *K. pneumoniae* liver abscess, even when an apparent biliary source has been identified (15, 16). These observations concern neoplasia and selected mucosal lesions; they do not establish that a small submucosal lipoma carries a similar risk or represents an equivalent source of PLA. In the present case, the hypervirulent phenotype of the *K. pneumoniae* isolate was not specifically tested and therefore cannot be inferred.

Several case reports and series have similarly described liver abscesses associated with benign or premalignant colonic lesions, particularly colonic adenomas (6, 10–12). These observations suggest that colonic lesions—despite their benign nature—may be associated with PLA, potentially through mucosal disruption and subsequent bacterial translocation; however, this mechanism was not demonstrated in the present case.

Table 1 summarizes selected published reports of PLA associated with benign or premalignant colonic or closely related gastrointestinal lesions. To the best of our knowledge, based on the literature search performed for this report, no previous case has specifically described PLA associated with an inflamed colonic lipoma.

Large colonic lipomas may present with areas of mucosal ulceration, ischemic necrosis, and local inflammation (17–19). This phenomenon has been documented in case reports describing ulcerated colonic lipomas that macroscopically and histologically mimicked malignancy (18, 19). These reports concern other patients and are cited here to provide context for the histopathological assessment of the present specimen. In our case, the overlying epithelium showed focal superficial abrasion/erosion opposite the lipoma, but deep mucosal ulceration was not demonstrated histologically.

The findings observed in the present case should be clearly distinguished from mechanistic hypotheses. The documented findings included a *K. pneumoniae* PLA without a CT-detectable biliary source, a sigmoid inflammatory lesion subsequently identified as a 1.2 cm submucosal lipoma, acute neutrophil-rich inflammation involving the mesocolic/subserosal tissues and extending into the muscularis propria, and focal superficial epithelial abrasion/erosion opposite the lipoma. By contrast, portal venous translocation originating from the lipoma remains hypothetical. An ulcerated lipoma could, in principle, share features with other mucosal lesions associated with PLA, including disruption of the mucosal barrier, exposure of deeper tissues to colonic flora, and local inflammation (20). However, in the present case, deep mucosal ulceration, ischemic necrosis, intramural abscess formation, microorganisms, and perforation or microperforation were not demonstrated histologically. Accordingly, the present lesion is described as an inflamed submucosal lipoma with focal overlying epithelial abrasion/erosion, rather than as a frankly ulcerated or infected lipoma.

Although most symptomatic colonic lipomas exceed 2 cm in diameter, lesion size alone is unlikely to determine the risk of bacterial translocation. Local mechanical factors, including intermittent torsion, transient intussusception, or focal ischemic injury associated with repeated mucosal trauma, may lead to mucosal injury in other reported lipomas (21, 22). Those mechanisms were not documented in this patient and remain speculative.

The presence of neutrophil-rich inflammation in the muscularis propria, subserosa, and mesocolon indicates an active inflammatory process in the resected segment. However, these findings do not, by themselves, establish that the lipoma was the primary infectious focus. Alternative or contributing explanations include occult adjacent inflammation, undetected microperforation, occult diverticular disease, other cryptogenic mechanisms, or biopsy-related injury. The prior endoscopic biopsies were described as superficial, whereas the resection specimen showed inflammation involving the subserosa and mesocolon. Nevertheless, the available pathological findings do not definitely exclude a biopsy-related or other iatrogenic mechanism. Colonoscopy was also performed after antibiotics had already been initiated, which may have modified the endoscopic appearance of the lesion. Importantly, no diverticula were observed at colonoscopy, and neither a diverticulum nor a perforation tract was identified in the available histological sections. Occult diverticular disease and microperforation therefore remain unproven possibilities rather than demonstrated findings.

The isolation of *K. pneumoniae*—a pathogen frequently implicated in liver abscesses of gastrointestinal origin—is compatible with hematogenous or portal bacterial dissemination from a possible colonic source; however, cryptogenic *K. pneumoniae* liver abscesses have also been described (23). Negative blood cultures, together with the absence of culture from the colonic lesion and the lack of microbiological or molecular comparison between the colonic lesion and the hepatic isolate, prevent confirmation of a common microbiological source. No microorganisms were identified in the available hematoxylin‒eosin-stained sections, and no special stains were performed.

This report has several important strengths, including a comprehensive diagnostic work-up integrating cross-sectional imaging, percutaneous drainage with microbiological analysis, colonoscopy, and definitive histopathological examination. Despite this evaluation, no alternative source of the pyogenic liver abscess was identified, leading to the consideration of the sigmoid lipoma as a possible, albeit unproven, colonic association.

Several limitations should be acknowledged. As a single case report, the coexistence of the sigmoid lipoma and PLA does not establish causation, and the lipoma may have been incidental. The colonic lesion was not cultured, nor was there any microbiological or molecular comparison with the hepatic *K. pneumoniae* isolate. A hypervirulent phenotype of *K. pneumoniae* was not assessed. Focal abrasion of the surface epithelium was identified opposite the lipoma, but deep mucosal ulceration was not demonstrated histologically. Only a limited number of histopathological fields were available for review; therefore, a focus of deeper ulceration, microperforation, or microorganisms elsewhere in the specimen cannot be entirely excluded, although none of these features was identified in the available sections examined. Special stains for microorganisms were not performed. The decision to perform surgical resection was based on the initial suspicion of malignancy rather than on evidence that the lipoma represented an infectious focus.

These limitations require that the association be described as possible rather than causal, and that the principal clinical message remain cautious: in patients with otherwise unexplained PLA, particularly when *K. pneumoniae* is isolated and no biliary or other common source is identified, evaluation of the colon, including colonoscopy, may be reasonable. This single case does not justify regarding small colonic lipomas as established infectious sources.

## Conclusion

An inflamed sigmoid submucosal lipoma with focal superficial epithelial abrasion/erosion was identified in a patient with *K. pneumoniae* PLA and no CT-detectable biliary source. Histological examination confirmed a mature 1.2 cm submucosal lipoma and acute neutrophil-rich inflammation of the colonic wall opposite the lesion, extending from the mesocolic/subserosal tissues into the muscularis propria. Deep or transmural mucosal ulceration, necrosis, intramural abscess formation, microorganisms, and perforation were not demonstrated in the available histological sections. The coexistence of the inflamed colonic lesion and the PLA is compatible with a possible colonic association but does not establish a causal relationship or confirm portal bacterial dissemination from the lesion. Infection of the lipoma itself was not microbiologically documented. Reported associations between selected colonic lesions and PLA support consideration of colonic evaluation, including colonoscopy, in otherwise unexplained cases, particularly when *K. pneumoniae* is isolated. These observations do not justify considering small colonic lipomas as established infectious sources.

## Data Availability

The original contributions presented in the study are included in the article/Supplementary Material, further inquiries can be directed to the corresponding author.
